# Challenges of implementing computer-aided diagnostic models for neuroimages in a clinical setting

**DOI:** 10.1038/s41746-023-00868-x

**Published:** 2023-07-13

**Authors:** Matthew J. Leming, Esther E. Bron, Rose Bruffaerts, Yangming Ou, Juan Eugenio Iglesias, Randy L. Gollub, Hyungsoon Im

**Affiliations:** 1 Center for Systems Biology, Massachusetts General Hospital, Boston, MA USA; 2 Massachusetts Alzheimer’s Disease Research Center, Charlestown, MA USA; 3 Department of Radiology and Nuclear Medicine, Erasmus MC, Rotterdam, The Netherlands; 4 Computational Neurology, Experimental Neurobiology Unit (ENU), Department of Biomedical Sciences, University of Antwerp, Antwerp, Belgium; 5 Biomedical Research Institute, Hasselt University, Diepenbeek, Belgium; 6 Boston Children’s Hospital, 300 Longwood Ave, Boston, MA USA; 7 Center for Medical Image Computing, University College London, London, UK; 8 Martinos Center for Biomedical Imaging, Harvard Medical School, Boston, MA USA; 9 Computer Science and Artificial Intelligence Laboratory, Massachusetts Institute of Technology, Cambridge, MA, USA; 10 Department of Psychiatry, Massachusetts General Hospital, Harvard Medical School, Boston, MA USA; 11 Department of Radiology, Massachusetts General Hospital, Boston, MA USA

**Keywords:** Medical imaging, Translational research

## Abstract

Advances in artificial intelligence have cultivated a strong interest in developing and validating the clinical utilities of computer-aided diagnostic models. Machine learning for diagnostic neuroimaging has often been applied to detect psychological and neurological disorders, typically on small-scale datasets or data collected in a research setting. With the collection and collation of an ever-growing number of public datasets that researchers can freely access, much work has been done in adapting machine learning models to classify these neuroimages by diseases such as Alzheimer’s, ADHD, autism, bipolar disorder, and so on. These studies often come with the promise of being implemented clinically, but despite intense interest in this topic in the laboratory, limited progress has been made in clinical implementation. In this review, we analyze challenges specific to the clinical implementation of diagnostic AI models for neuroimaging data, looking at the differences between laboratory and clinical settings, the inherent limitations of diagnostic AI, and the different incentives and skill sets between research institutions, technology companies, and hospitals. These complexities need to be recognized in the translation of diagnostic AI for neuroimaging from the laboratory to the clinic.

## Introduction

Computer-aided diagnostic (CAD) models are computer algorithms capable of making a prognosis or diagnosis about the health of a patient, given available data. CAD models for radiological images have been widely applied in breast cancer screening in mammograms^1,2^, largely to automate repetitive tasks, and, more recently, AI tools for the detection of intracranial hemorrhages (ICH) and large vessel occlusion (LVO) in CT images have been approved by the FDA and validated in further studies^3–5^. The eventual, widespread clinical application of CAD models^6^ to brain images routinely collected in hospitals, such as CT and MRI, holds promise to automate the diagnostic process, reduce rates of misdiagnosis of brain-related disorders^7–10^, reduce diagnostic wait times^11,12^, cut costs, increase diagnostic objectivity^13^, and inform doctors in their assessment of patients^14^ for a wide range of brain disorders. Decades of research in machine learning—accelerated in recent years by the surge of interest in deep learning—has led to developments in the research world of CAD models for brain images across a wide range of psychological and neurological disorders^15–17^. In spite of this, however, very little systemic, real-world, clinical translation has thus far occurred^18^. This is not entirely unexpected, given historic trends. Oakden-Rayner^6^ describes the history of computer-aided detection in radiology as well as its disappointing results in the initial waves of AI, specifically for mammography diagnosis^2,19–21^, given the limited ability of early diagnostic models. His article provides, in contrast, a more optimistic light on current CAD models because of deep learning’s unprecedented success in other areas of science. This success, however, does not guarantee that it can be implemented successfully in healthcare because success in healthcare is only partially related to the reported efficacy of CAD models.

In this article, we attempt to characterize the ongoing progress and future directions of CAD models in translational neuroimaging. We first review the development of CAD models in the research world, covering the continuum of methods with current clinical applicability, those under active development, and those with potential future applications. We then discuss the general challenges of developing CAD models from a purely technical perspective, including issues both unique to healthcare and those seen in machine learning generally. Finally, we discuss translational pathways for bringing neuroimaging CAD models to the clinic as well as the institutional, cultural, and sociological barriers that affect health AI research more generally. We end by suggesting potential future directions and scenarios for translating diagnostic AI to the clinic.

## Utility of CAD models for brain images currently being developed in research settings

**Fig. 1:**
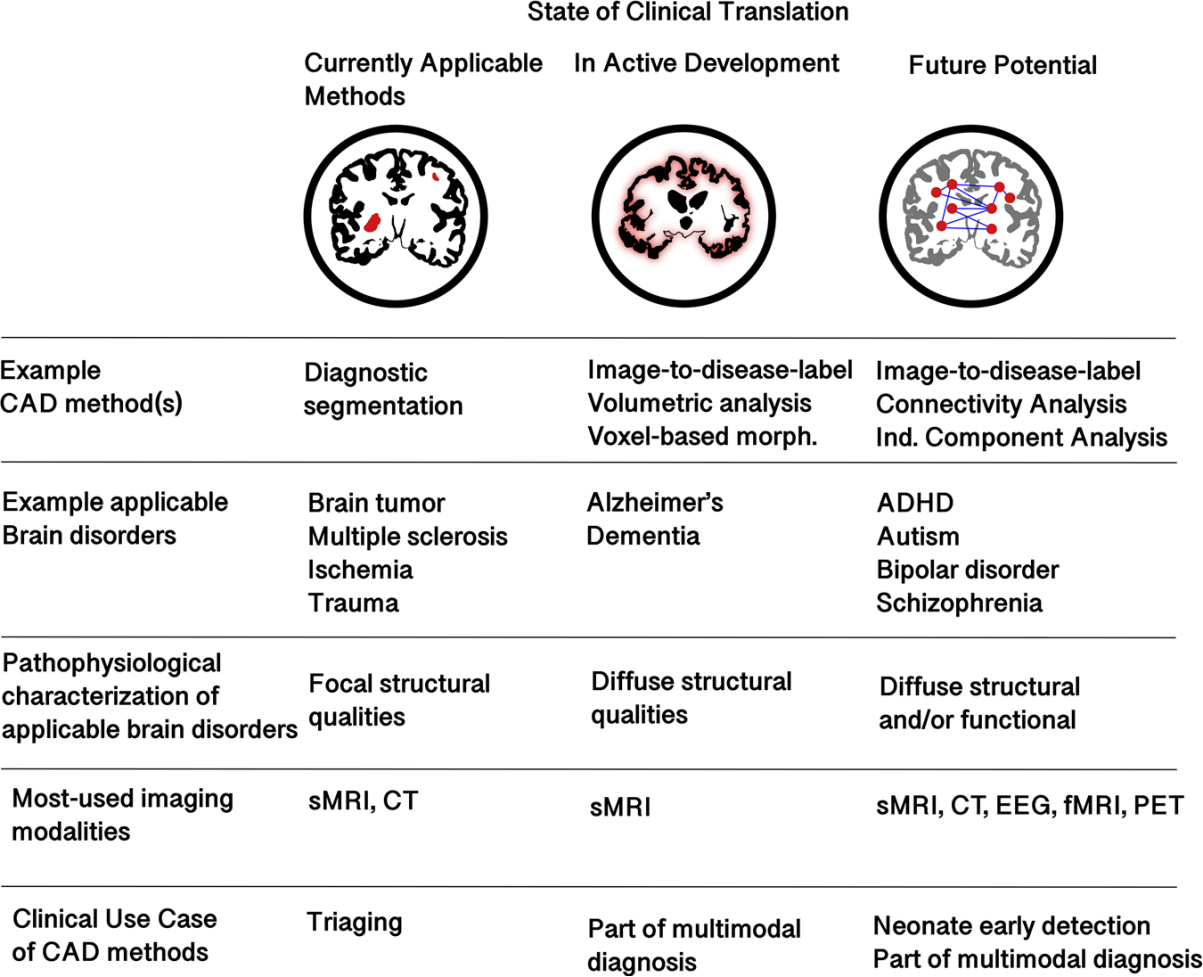
Current development of different types of neuroimaging CAD models. Neuroimaging CAD models and analysis methods exist on a continuum of development and clinical applicability. Models that use diagnostic segmentation can be applied to brain disorders characterized by focal structural anomalies, and they are in a better position today to be applied clinically. CAD models that output a label directly can help in diagnosing neurodegenerative disorders, which have an explicit, though diffuse, structural basis, and thus CAD models can be used to detect and inform their diagnosis. However, they have yet to see widespread clinical use or a specific clinical need. Brain disorders characterized by both diffuse structural and functional qualities have been analyzed by CAD models, but specific biomarkers are elusive and their clinical implementation would require further development.

Several past reviews have focused on the development of CAD models for the diagnosis of different brain-related disorders (such as Alzheimer’s disease, Parkinson’s disease, and multiple sclerosis) based on radiological images^15–17,22^. This work has shown that these disorders exist on an evolving continuum and vary in terms of CAD models’ ability to detect them in neuroimages, neuroimaging modalities required to detect them, and potential clinical use cases of such models. For the purposes of this review, we present a cursory overview of these findings in three very broad categories to facilitate making our key points: those with current clinical applicability, those under active development, and those with future potential. As we will see, these three categories generally include, respectively, brain disorders characterized by explicit lesions, neurodegenerative disorders characterized by diffuse structural qualities (these qualities include both normal and abnormal image features), and psychiatric disorders that are characterized by both diffuse functional and structural qualities. These arbitrarily defined categories of the continuum are summarized in Fig. 1.

### Neuroimaging CAD models with current clinical applicability

We first look at neuroimaging CAD models with current clinical applicability. As of April 2023, the FDA has approved 82 neuroradiology AI models for clinical use^23^. These fall into three categories: Medical Image Management and Processing System (MIMPS), which is software used to preprocess and manage radiology images; computer-aided triage and notification (CADt) models; and computer-aided diagnosis (CADx). As of April 2023, 58 of these are MIMPS, 22 are CADt, and 2 are CADx. While many MIMPS models may rely on AI, they are not used directly for making a diagnosis. CADt/CADx models could be useful in the general analysis of neuroimages—e.g., for quantifying the volume of specific brain regions, which could help clinicians in the diagnosis of dementia. In particular, FDA-approved models for the triaging of structural CT images^3–5,24^ are used to detect brain disorders that are characterized by a local, structural anomaly that can be seen by a human expert.

It is already routine clinical practice to detect suspicious lesions based primarily on radiological images. As a result, the modalities used to acquire them, namely CT and different forms of structural MRI, are already in common clinical use. A range of disorders is characterized by such focal structures, including brain tumors^25,26^, multiple sclerosis lesions^27–29^, and various forms of traumatic brain injury^12,30^ such as intracranial hemorrhage^11^, intracranial mass effect, and stroke^31^.

From a technical perspective, AI models that analyze such localized disorders are distinct from the detection of diffuse functional or structural disorders, covered below; the detection of tumors, hemorrhages, or structural damage by traumatic brain injury may use a segmentation algorithm that can be verified visually (or a simple binary detection algorithm that is nonetheless easy to verify), and the presence of visible biomarkers are present by definition. In contrast, the more sophisticated CAD models that translate brain images directly to a diagnostic label (as opposed to segmentation-based models) generally lack a visually interpretable output and are thus more difficult to validate. Success in disease detection is largely reflected in the literature. Recent models are variously able to find tumors, regardless of the specific type of tumor, in MRI at higher than 95% accuracy in the most recent studies^32^; intracranial hemorrhages in CT at rates ranging from 82% to 96%^33–35^; and multiple sclerosis lesions, as measured by dice similarity coefficients, between 0.35 and 0.95^36^. (Note that sensitivity and specificity, or AUROC, are more preferred performance metrics than accuracy^37,38^, though we compare accuracies here since those are most commonly cited across studies, especially those that are older). Each of these methods is highly dependent on the dataset used and the specific method of measurement, but the emerging picture is that they are remarkably effective in the laboratory.

**Fig. 2:**
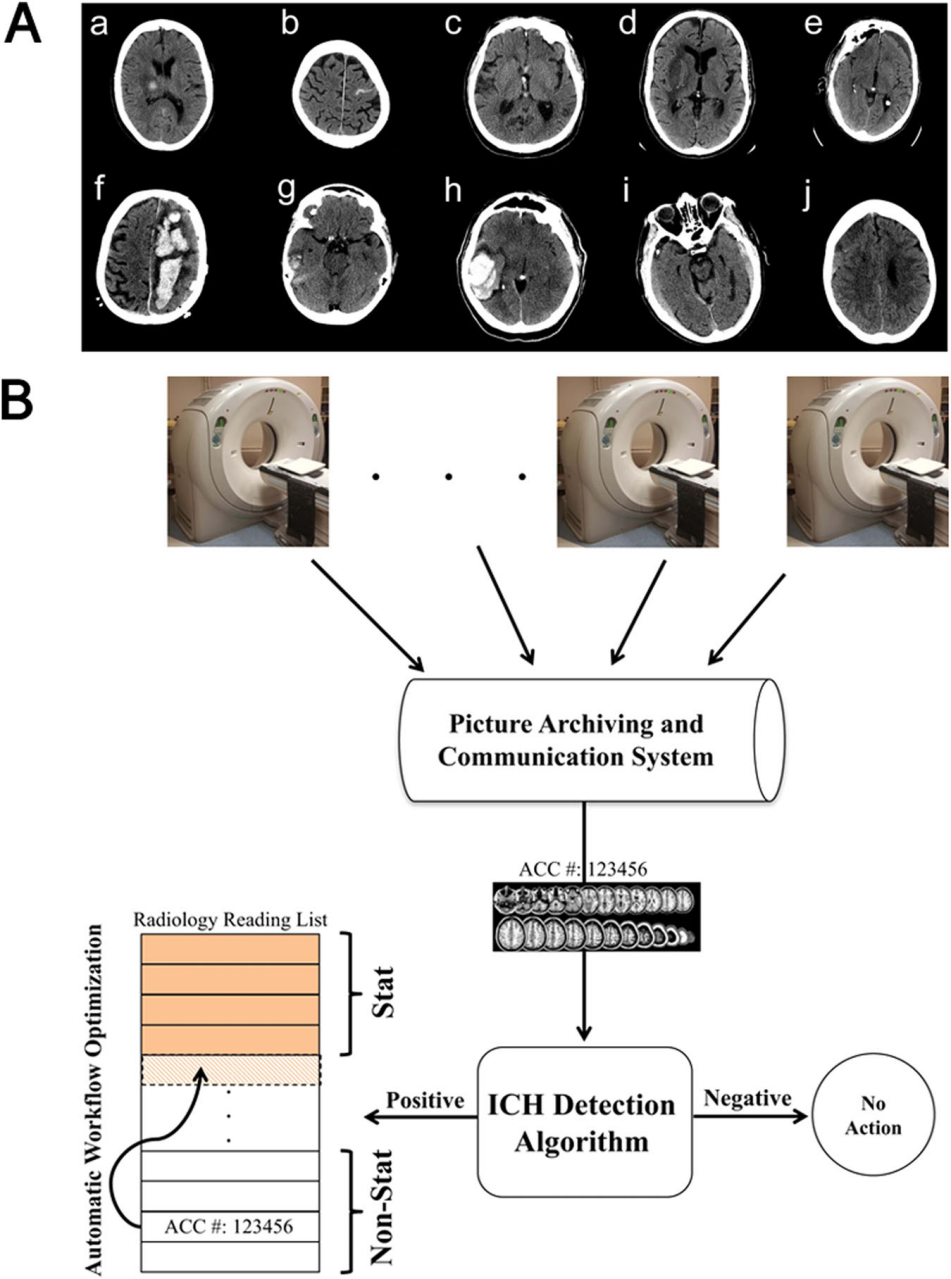
Workflow integration of ICH detection model for brain CTs, from Arbanshirani et al.^11^. **A** Head CT image with ICH. **B** A clinical workflow integration of a triaging tool that detects ICH automatically and subsequently reclassified it from “routine" to “stat", which significantly decreases the average time required to detect ICH. This is one of the few cases of a neuroimaging CAD model being integrated into clinical workflow to address a specific clinical need. We would like to thank Aalpen A. Patel for permission to use figures in this paper.

For clinical translation, however, such models need to be validated for specific clinical needs. In a unique report of a neuroimaging CAD model being implemented and validated clinically, Arbabshirani et al. proposed the use of an intracranial hemorrhage detection algorithm for CT scans at the Geisinger Department of Radiology in Pennsylvania to evaluate routinely collected CT scans^11^. The model, which was trained on historical data collected at Geisinger, triaged data. It reclassified those scans in which intracranial hemorrhages were detected from “routine” to “stat”, meaning that they would be prioritized for clinical evaluation much sooner than normal. This is one of the only cases of a neuroimaging CAD model being integrated into a clinical workflow (Fig. 2). Notably, in favoring higher sensitivity and lower specificity, the model did not have to work perfectly for it to be clinically useful since it was designed to accelerate the overall decision-making process by passing on likely images to human interactors rather than making a final decision (decreasing the median diagnostic time from 512 min to only 19 min). This established a concrete clinical use case for this particular neuroimaging CAD model, which was followed by several algorithms designed to detect ICH and LVO in CT images^3–5^. As mentioned above, these are the only class of disease-detection AI algorithms for brain images currently approved for clinical use by the FDA^24^.

Triaging can be applied as well to the detection of traumatic brain injuries, since CAD models’ fast and automatic analysis can make them useful^12^, and their clinical effectiveness in minimizing the lead time delay to treatment could be further tested. Detection of focal structural features of other diseases may not have a similar time-sensitive clinical need to drive clinical adoption. Another valuable use case of these models is to standardize clinical review. In this case, however, the high-sensitivity paradigm described above would only in part meet clinical needs, and models would require further validation to ensure clinically meaningful specificity. This includes validation in prospective large cohort studies to assess the clinical feasibility of the models in comparison with readouts from expert radiologists and the general applicability of a given imaging instrument, especially for abnormality detection in local clinics where expert radiologists may not be readily available.

### Neuroimaging CAD models in active development and testing

We next consider models that are under active development and for which widespread clinical implementation is possible, but still elusive. Unlike those disorders covered above, there are neuropsychiatric disorders for which specific and focal anomalies have not yet been established as accepted biomarkers but have established clinically meaningful correlations between available imaging features of brain structure and behavior^39^. Diagnostic algorithms for local abnormality detection, such as those described above, are less applicable to these. Thus, another class of analysis techniques is often used. Direct image-to-label methods, for instance, can be applied for disease detection, even in routinely collected clinical data^40^. Other methods, such as voxel-based morphometry (VBM)^41^ or forms of volumetric analysis like cortical thickness measurements^42^ do not output a prediction directly but would be particularly useful in the diagnostic process because they focus on the quantification of a wide range of diffuse effects, such as subtle changes in gray matter volume, local diffusion properties, cortical thickness, and the shapes and signal intensities of structures in multimodal MRI scans^43^. This analysis is more difficult for unaided humans to accomplish than the identification of localized structural lesions.

In particular, CAD models for neurodegenerative disorders, namely Alzheimer’s disease on structural MRI, is widely studied in the research world^22,44^. Alzheimer’s is a particularly common disease^45^, and early diagnosis could have a substantial impact on patient well-being and advance care planning^46^. It also has an established association with brain structure^47,48^, and has several publicly available datasets^49–51^ on which CAD models can be developed and tested. Interest in finding imaging biomarkers for Alzheimer’s disease is largely centered on the need for both early detection and differential diagnosis, or the need to distinguish AD from other underlying causes of dementia. Commonly intended outputs of such models may be an exact diagnosis, a prognosis, or, more accurately modeling the disorder, and its temporal progression^52^. Even outside of diagnosis, such temporal progression models may expand on the clinical diagnostic models discussed in this article by, for instance, detecting and selecting appropriate at-risk subjects for clinical trials, greatly reducing sample sizes^53^.

The use of structural images as one factor in reaching a diagnosis of Alzheimer’s is common clinical practice^47,54^, but, in this area, analysis of neuroimages is often done without the use of the quantitative algorithms that are often studied in the research world^22^. The slowness in adopting such methods to aid in this diagnosis, in spite of the interest in the development of CAD models in research^22^, has been the subject of some recent interest^54^ and is discussed further below.

Why have these methods had difficulty finding a place in routine clinical practice? One obvious reason is that, while CAD on Alzheimer’s is often studied in research settings, much of the literature on neuroimaging in degenerative disorders is largely inapplicable to clinical CAD models because so many of the studies focus on average group differences, reporting the significance of a particular statistical test, rather than single subject classification^55^, which reports metrics such as sensitivity, specificity, accuracy, and AUROC. For example, while there is an average group difference between AD patients and controls in hippocampal volume in large cohort studies, the inter-individual variance in hippocampal volume renders that metric alone insufficient for diagnostic utility. Thus, much of the research into particular disorders, while insightful for understanding them generally, is of limited utility when designing single-subject diagnostic models.

The substantial corpus of literature reporting on single-subject CAD models to detect Alzheimer’s disease demonstrates the enthusiasm for this approach, even as success remains elusive. Studies of Alzheimer’s detection in public datasets have reported a very broad range of accuracies, ranging between 58% and 100%; however, critical issues such as data leakage and cross-dataset generalizability were unaddressed in many of these studies, which could have led to poor outcomes^22,56,57^. Differences with respect to the use of a single MRI sequence (e.g. T1-weighted structural MRI) versus using multiple sequences and/or imaging modalities have an impact on the CAD model outcomes, as does the classification task (e.g. distinguishing between AD, different forms of Mild Cognitive Impairment (MCI), control groups, or a combination thereof). When considering only those studies that compared AD to controls, did not have any detected data leakage, and classified using a 3D CNN on a subject level, between 76% and 90% classification accuracies were reported^58–62^, with the average being 83.4%^22^, providing some confidence that this approach is making a useful contribution to disease detection. (Note, again, that sensitivity and specificity, or AUROC, are more preferred performance metrics than accuracy^37,38^, though we compare accuracies here since those are most commonly cited across studies, especially those that are older).

An approach that might significantly speed the path to clinical implementation would be the use of CAD models that use as inputs more information about the patient than just the structural brain image^63^. Such multi-input models have been studied^64,65^, but they are relatively rare. This is likely due to both the lack of detailed, structured, demographic, and clinical data in public imaging databases and the fact that off-the-shelf, image-to-label machine learning models are common in computer vision and thus are easier to implement than multi-input models. Vinters et al.^47^ noted five accepted methods of diagnosing Alzheimer’s disease, with structural neuroimaging being only one; thus, the very task of attempting to diagnose Alzheimer’s from structural MRI immediately makes CAD models for radiology weaker than the multi-input diagnoses that can be utilized by a clinician. This suggests that in order to achieve clinically relevant performance, multi-input diagnostic CAD models ought to be utilized; this is discussed further below, in Section ‘Challenges in designing robust CAD models for the clinic’.

Separating the current research perspective further from clinical reality, differential diagnosis is also understudied in single-subject classification, although notable exceptions exist^66,67^. Lack of information about the potential of CAD models to contribute meaningfully to a clinician who needs to formulate a differential diagnosis for a patient presents another translational complication. Data in single-subject classification studies often come with binary or categorical labels between healthy controls and different stages of Alzheimer’s. However, the clinical question is less often about whether a patient has Alzheimer’s or is cognitively normal, but whether they have a prodromal stage of Alzheimer’s or whether they have another, non-Alzheimer’s-related neurological disease (e.g. vascular or frontotemporal degeneration).

Even with all these caveats, however, such models and methods do show promise for eventual clinical translation, especially as scientific studies using advanced neuroimaging technologies that reveal the pathobiological mechanisms underlying disorders become more relevant to day-to-day clinical practice.

### Neuroimaging CAD models with potential future applicability

We now consider neuroimaging CAD models that may have potential future clinical use, but which for multiple reasons, need further development. These are CAD models and techniques used to analyze brain disorders for which there are not yet any confirmed pathophysiological mechanisms or consistent imaging biomarkers. Studies reporting on CAD models for these applications often use imaging techniques that, as a consequence of this, are not a component of common clinical practice in the care of these patients. This is the case for a wide range of neuropsychiatric disorders that have been characterized in research settings by evidence of both diffuse structural and functional neuroimaging abnormalities. A huge body of neuroimaging research has focused on psychiatric disorders^15,16^, such as autism^17^, schizophrenia^68–70^, autism spectrum disorder (ASD)^71–75^, bipolar disorder and depression^76,77^, and attention deficit and hyperactivity disorder^78–83^, to name just a few. Such disorders are typically diagnosed using clinical interviews and behavioral tests^84^, but these combined with neuroimaging-based and other quantitative, objective biomarkers^85^ would help to make a more objective assessment and inform classifications of particular psychiatric disorders.

CAD models based on neuroimaging for the detection and diagnosis of psychiatric disorders are promising, but the clinical translation may be a long way off. Like neurological disorders, literature on psychiatric disorders typically shows widespread associations between brain structure^75,86^ and function, and they are seldom completely characterized by localized structural changes that would be readily identifiable by a practicing radiologist or other clinicians. In addition, because the association between brain structure and behavior is weaker due to limitations in the sensitivity of the imaging techniques or a pathophysiological mechanism that has no impact on brain structure, many psychiatric disorders are primarily studied using modalities that are not yet routinely acquired in clinical practice and which themselves require unique technical analysis methods; in particular, functional brain activations, recorded with EEG or fMRI, are more often collected for research studies than clinical diagnostics. These are studied using the methods described in Section ‘Neuroimaging CAD models in active development and testing’, but also a range of more complex data analytic techniques, such as independent component analysis (ICA)^87^ and different forms of brain connectivity^88^.

Another technical obstacle is that the models often require imaging modalities that are not currently implemented in clinical practice; this is not usually acknowledged in the literature describing research aimed at elucidating disorder-specific brain imaging biomarkers. Thus, single-subject diagnostic models for psychiatric disorders developed in laboratory settings would not be immediately translatable. The American Psychiatric Association published a report concluding just as much in 2019^84^, which noted, in studies that attempted to find structural biomarkers for single-subject classification, inconsistent regions of interest and a requirement of at least 80% sensitivity and specificity for the diagnosis or detection of adult mood and anxiety disorders, psychotic disorders, cognitive disorders, substance use disorders, and various childhood disorders, including attention deficit hyperactivity disorder, childhood bipolar disorder, depression/anxiety, and an autism spectrum disorder. Thus, for the clinical implementation of CAD models to aid in the detection or diagnosis of psychiatric disorders, either additional imaging modalities would need to be clinically implemented, or CAD models for structural neuroimages would need to significantly improve.

Like the neurological disorders discussed above, clinical applications of CAD models to assist with other tasks such as the generation of differential diagnoses are not as often the focus of single-subject neuroimaging CAD studies for psychiatric disorders. There is ample room for expansion of the focus of research to address unmet clinical needs. However, in the present, a particularly potent clinical use case for the detection of psychiatric disorders is neonate imaging, because behavior assessment in the neonatal stage is challenging. Studies in CAD models for neonate imaging have shown the ability to predict arbitrary factors, such as brain age and myelination^89^, as well as success in predicting familial risk for autism spectrum disorder^90^ and subtle brain injury^91^. While the acquisition and analysis of neonate images present unique technical challenges^92^, the genetic basis for many such disorders^93^ and the potentially huge impact of very early intervention^94–96^ makes early detection a strong use case for CAD algorithms for psychiatric disorders.

## Challenges in designing robust CAD models for the clinic

As just reviewed, neuroimaging CAD models have seen some successes in controlled laboratory settings, but unique technical and disease-related challenges, often not reflected in the laboratory studies, hamper their translatability. These are summarized in the first two sections of Table 1 and are discussed in greater detail here.

**Table 1:** A breakdown of the barriers towards clinical implementation of neuroimaging CAD models presented in this article.

Barrier type	Reason	Description	Solution(s)
Technical	Generalizability	Failure of CAD models to generalize across different scanner types and hospitals, as well as different population subgroups, ethnicities, ages, and genders	Federated learning, larger datasets, methods that prevent overfitting, domain switching, harmonization
	Verifiability	A general set of problems, including the black box model, prevents users from knowing the reasons for a CAD model’s decision.	Segmentation-based models explainable AI, gradient class activations
	Integration into workflow	Translation of models from proof-of-concept to usable software products	Investment in software engineering and user experience, corporate partnerships
	Incomplete and mislabeled data	EHR data is often incomplete or mislabeled, hampering the training of CAD models	More careful record keeping, translating clinician notes, careful exclusion of data, and development of methods that can handle such incomplete data.
	High computational requirements	Computational requirements for medical image computations are very high, which is expensive.	Cloud-based solutions; institutional investment in servers
Disease-related	Lack of biomarkers	Lack of consistent physiological features detectable in data that are consistently present with a particular brain disorder	Dependent on the type of disorder studied, and for some it may be insurmountable. However, higher-resolution data, different modalities, and more advanced analysis techniques may mitigate the issue.
	Lack of sufficient modalities	Modality types used in the research world (primarily to study psychiatric disorders) are often not present in the clinic, curtailing the implementation of neuroimaging for the detection of such disorders	Inclusion of fMRI, EEG, etc. into clinical workflows
	Disease differentiation	Emphasis on causes of the disease (e.g. whether dementia is caused by Alzheimer’s or vascularization), which is often just as important as the presence of a disease	More careful labeling of disorders and confounders, further study of ML methods beyond binary classification
	Correlation with confounding variables	Variables for which the disorder of interest is systematically correlated with another variable regardless of the dataset; similar to generalizability (above), except different methods are required to mitigate model bias	Data matching, machine-learning-based regression methods
	Lack of control group	Clinical data often lacks a healthy control group, against which to compare, to train CAD models	Careful data curation; reformulation of the problem, such that a control group doesn’t have to be healthy, but merely has to not have the disease of interest
Institutional	Separation of AI experts and data scientists from clinicians	Data scientists and AI experts are most often employed at sites other than hospitals, thus being separated from real-world medical data, while clinicians work in hospitals, leading to incomplete understanding on both sides	Increased postdoctoral salaries in research hospitals, stabilization of career tracks for junior biomedical researchers, specialized fellowship programs to partner AI experts with clinicians
	Technical expertise of clinicians/Usability of CAD models	Clinicians are disinclined from using CAD models and other automated tools due to the technical skill required and the amount of time required for use	Work more closely with data scientists/AI experts, supplementary training courses; Prioritization of usability in CAD models
	Lack of funding for implementation studies	Funding bodies are often more inclined to fund novelty studies rather than implementation studies	Different guidelines for funding bodies (e.g. NIH)
	Disorganization of clinical databases	Related to “incomplete and mislabeled data," above. Databases in hospitals are often disorganized, hampering big-data machine-learning studies and leading to mislabeled data. Medical images are often duplicated and identifiers are often missing or difficult to match with medical images, leading to loss of clinical/demographic information for medical images.	Institutional investment clinical databases, both on the part of hospitals and vendors.
	Federal approval processes	Federal bodies are often disinclined from approving CAD models, though much of this is a result of the above issues	Addressing many of the above problems, leading to greater confidence in the efficacy of CAD models; clarification, on the part of FDA and other regulatory bodies, of requirements for CAD model implementation and approval
	Underdeveloped business model of medical AI	Lack of development of business model for medical AI. Who does the value accrue to, and who pays for it?	Development of AI business models in other industries and in business schools, which will likely inform the best practices for doing so in medicine.
	Lack of capabilities for post-market surveillance	After an AI model is implemented in a hospital, what mechanisms are available to monitor their effectiveness on a large scale?	Centralized monitoring and reporting systems that do not interface with patient data directly, thus ensuring security.

Technical- and disease-related challenges are discussed in the section “Challenges in designing robust CAD models for the clinic”, and institutional challenges are discussed in the section “Pathways to clinical implementation and institutional barriers”.

In current practice, neuroimaging CAD models are most often tested on small datasets acquired for a specific research study or large public neuroimaging benchmark datasets, both of which are usually collected on a limited number of very similar sites with consistent diagnostic techniques. However, this does not reflect the substantial differences in manufacturer, quality, and clinical practices often found in real-world hospitals. For instance, the UK Biobank, a widely employed public imaging benchmark dataset that includes brain MRI scans for what will be a total of 100,000 participants, restricts image acquisition to four scanning sites^97^, each of which has identical scanner hardware and software, and performs regular quality checks to ensure the harmonization of the image data. In stark contrast, our large-scale study of brain MRI data from of 37,311 patients extracted from the clinical archives of a pair of academic healthcare centers over a 25-year period^98^ was collected on 954 unique scanners, reflecting the much more diverse array of technical confounds in real-world data.

These clinical site differences, a manifestation of the common machine learning problem of dataset shift^99^, have significant implications for CAD models, especially ones trained using machine learning. Importantly, while machine learning models have shown the ability to *generalize* across a complex dataset, they fail to *extrapolate* even simple mathematical functions; thus, an input much higher or lower than what is found in the training set would break the model^100^. This would make CAD models potentially unreliable if any single parameter that lies too far outside the extremes of its training set is included. The same could be true of a combination of specific parameters not seen in the training set. This would also mean that rare diseases or images acquired on new scanners may introduce wholly unseen variations and thus make the models even more likely to fail. This issue has even led the FDA to allow automatic updates of AI/ML products for issues such as equipment upgrades and changes^101,102^.

A number of technical and strategic methods have been proposed to overcome the site difference problem. It may be mitigated, to an extent, by careful quality assurance and clinician oversight of any new data, with human experts assuring that no inputs to a CAD model are too flawed or different from the training data. Given the busy environment and the amount of required knowledge and tasks, however, this presents a logistical barrier in itself. New sophisticated approaches for automatic quality control strategies have been suggested^103–105^, and leveraging them in parallel with expert review will make the quality assessment process more efficient.

Further generalization of CAD models may also be achieved by an expansion of the training set, but health privacy laws and the expense of acquiring brain images limit the ability to use this strategy. In healthcare particularly, federated learning^106,107^, in which models are trained internally in several different sites for generalizability, has great potential to be an effective method to improve model robustness that also follows data privacy laws. Recent advances are supporting and enabling this approach^108,109^. Adversarial deep-learning regression methods^110^ as well as simpler statistical regression methods such as ComBAT^111,112^ for multi-site MRI harmonization have been shown to be effective across public benchmark datasets like AIBL, ADNI, and ENIGMA. Such methods, however, have not been rigorously tested in the less-consistent environments of clinical neuroimaging. Keenan et al.^113^ measured differences in T1 MRI scanner measurements among 27 different configurations of manufacturer, software, and field strengths on phantom data and found substantial and inconsistent discrepancies between MRI scanners, suggesting site difference regression would be more challenging across random hospitals than across benchmark data. Some early attempts to address this have relied on generalized domain-switching deep learning models, namely SynthSeg^114^ with evidence that this approach has meaningful clinical validity^115^.

Similar to site differences in their ability to disrupt the effectiveness of CAD models are confounds systematically associated with brain disorders. These are measurements in which biomarkers are related to a given variable that ought not to be associated with a certain disease. The most obvious example of this is age and degenerative disorders, which would disrupt the detection of rare early-onset cases. Methods that simply generalize a model across different sites and populations, such as federated learning, would not sufficiently address such confounds. However, other strategies, such as ComBAT, post-hoc dataset matching^98^, and adversarial confound regression^110,116^ may still aid with these issues.

Even with the above issues addressed, however, differences between laboratory and clinical settings would further complicate, if not inherently prevent, any models trained and validated on research datasets from being deployed directly to hospitals. Gollub et al.^117^ describe four key differences between data acquired in laboratories and clinical settings: (1) acquisition (standardized versus according to the needs of the patient); (2) quality of data; (3) lack of a consistent control group in clinical data; and (4) reporting methods (quantitative reporting in research versus qualitative reporting in clinics). Many of these differences could conceivably be addressed by careful analysis of electronic healthcare record data by clinicians and data scientists. A team of clinicians and data scientists could translate qualitative reports to quantitative data on which to train CAD models, carefully partition and analyze electronic health records to isolate disease labels and associated training data, and so on. However, the issue of data quality would necessitate the re-evaluation of models entirely, since diagnostic results produced on high-resolution structural neuroimaging data are not at all guaranteed to replicate on low-resolution data. Lack of a healthy control group and disease-based confounds, as well as incomplete data^118^ that may fail to even note confounding pathologies and cause the model to be completely wrong, are all further reasons that models trained and validated on a research sample would need to be re-trained and re-evaluated within the unique context of a hospital.

As discussed above, clinically implemented CAD models for neuroimages, especially those targeted towards neurodegenerative or psychiatric disorders, would most likely need to take as input more information than just the brain image itself. The main reason for this is labeling. While most machine learning models were designed for tasks that require reliable, ground-truth labels, labels for CAD models in healthcare, especially for brain disorders, are often not obtained from the data themselves, but from other sources, such as behavioral assessments, prescribed medications, laboratory results, or other biomarker analyses, or even neuropathology. This often causes labels found associated with the medical image data alone to be wrong^119^, incomplete, or unrelated to the underlying biology. While other machine learning methods attempt to replicate the human performance, diagnostic AI on a given brain image is thus attempting to perform tasks that clinicians themselves do not perform (diagnosis from a structural MRI or CT scan alone), and so there is no verification that such a diagnostic task is even possible. This important difference between general machine learning and CAD models can be generalized to most psychiatric and neurodegenerative disorders studied in the neuroimaging literature, favoring the implementation of multi-input CAD models for the majority of clinical use cases^64,65^.

## Pathways to clinical implementation and institutional barriers

Thus far, we have focused on purely technical barriers to neuroimaging CAD model design and translation. We now turn our attention to institutional, cultural, and sociological barriers that impact the development of these tools and their translatability to clinical practice. These challenges are summarized in the bottom half of Table 1.

The bulk of research has been in the development of CAD models in research settings, but in recent years, cohorts of researchers around the world have been working to flesh out a full pathway to clinical implementation. Goodkin et al.^120^ describe the quantitative neuroradiology initiative (QNI) framework, a step-by-step process for the validation of neuroradiology quantification techniques for clinical practice, which is the most comprehensive and general framework to date for doing so. Briefly, it consists of six steps: (1) identify appropriate and proven imaging biomarkers for the disease in question and establish a clinical need; (2) develop and test an algorithm for automated analysis of these biomarkers; (3) communicate results in a quantitative report; (4) technically and clinically validate the algorithm; (5) integrate the algorithm into the clinical workflow; and (6) perform the in-use evaluation in the clinic.

A large number of studies attempt to achieve the biomarker identification of step 1, though few have been consistent over most brain disorders, as noted in First et al.^84^. Just as important but less often discussed is the true clinical need, as discussed extensively above, which is often unaddressed. Many studies have achieved steps 2 and 3 and the technical validation of step 4, showing highly effective CAD models in neuroimaging, but few of these have even attempted the clinical validation and workflow integration of steps 4 and 5, and even fewer have reached step 6. This is partially because clinical validation requires a different set of skills and resources than does purely technical development, which may only require access to computing resources and a public imaging dataset. Model robustness is difficult to prove without access to clinical data or knowledge of clinical workflow. Furthermore, in these studies, more thought is typically given to model uniqueness and test set accuracy than is given to valid clinical uses of the proposed model.

After all these steps are achieved, government approval, especially in the United States^121^, is a difficult process. Goodkin et al.^120^ note the difference between the rollout of such a model at a particular institution (which may be sufficient for QNI steps 4–6) and general approval of the medical product for all institutions in a given country. While models may be tested internally at a single institution^11^, in the United States, the Food and Drug Administration is the body responsible for the approval of such models prior to general use. The FDA’s own caution around CAD models was confirmed in a 2012 report, in which they considered computer-aided diagnosis to be higher risk than computer-aided detection^6,24,122^. A recent review by Khunte et al.^24^ reported that, of the more than 150 medical-imaging-related AI algorithms approved by the FDA to date, only three were related to brain MRI, and those applications were not CAD models, but, rather, were for perfusion quantification and segmentation; a more comprehensive database is maintained at https://aicentral.acrdsi.org/^23^. To date, the only truly diagnostic CAD algorithms (i.e., which output an estimated diagnosis) for neuroimages that have been approved by the FDA rely on similar local area detection for ICH and LVO identification^3–5,24^, after its unique success in single-institution clinical validation^11^, discussed in Section ‘Neuroimaging CAD models with current clinical applicability’ above. This abundance of caution on a federal level may be one of the impediments to the general rollout of CAD models (in U.S. hospitals particularly), but it is more likely a reflection of the difficulty of designing and validating effective CAD models, especially for brain images.

Even after government approval, continued monitoring of AI algorithms in the clinic is necessary, since changing clinical conditions or device upgrades may cause them to operate unpredictably in the future (as mentioned in Section ‘Challenges in designing robust CAD models for the clinic’, this motivated the FDA to update its AI/ML device guidelines to allow automated model updates^101,102^). Daye et al.^123^ recommended internal institutional governance committees that would both approve and require continual oversight of such algorithms throughout their lifespan.

Specific standards for CAD adoption and implementation vary widely from country to country. For a few examples, among digital health products, China considers those that rely on AI to be the highest-risk class^124^ and specifies that applicants must carry out a number of risk-management tasks to validate their AI algorithms prior to implementation. In India, comprehensive digital health laws are lacking, and the question of health AI models is generally unaddressed by their government^125,126^. Europe, despite not having a single regulatory agency across the continent, seems to have produced the most comprehensive academic thought surrounding CAD implementation, given its invention of QNI^120^.

While the primary reason for the lack of clinical adoption of CAD models is the lack of evidence that they can address key unmet clinical needs, it is not the only reason. The lack of progress in the rollout of neuroimaging CAD models is partially due to a lack of incentive to adopt them on the part of clinicians, which is both related to the usability of common tools and simple time constraints. One area where this has been studied is dementia diagnosis, specifically in memory clinics in Europe, where medical devices are not regulated by a single regulatory agency^121^. Few groups have published the results of the clinical implementation of tools validated under the QNI framework in clinical practice^127^. Vernooij et al.^54^ surveyed typical clinical practice in diagnostic radiology from 193 European academic and non-academic institutions, with 90% stating that they acquired some form of MRI for dementia diagnostics; 75% of centers used visual rating scales (i.e., in which radiologists analyzed the MRIs themselves), though only 5.7% regularly used volumetric data in their analysis. The most commonly cited reasons for their non-use were lack of access to algorithms and the additional time required to use them (a sentiment echoed in computer detection systems for mammogram diagnostics^128^).

Other impediments are related to market forces that affect health AI in general and are not specific to neuroimaging CAD models. Large, private tech companies that hire skilled engineers capable of designing these diagnostic AI algorithms have attempted to translate their health AI work into a clinical setting. To date, no major breakthroughs have been shared and a few of these high-profile projects have ended with little more than poor publicity, discouraging future endeavors. Concerns with data privacy and insufficient communication with regulatory bodies derailed Google’s Project Nightingale^129^ as well as DeepMind’s kidney injury detection algorithm^130^ with the NHS in Great Britain, while algorithmic efficacy, stemming from a lack of data, led IBM to close IBM Watson Health^131^. While these failures have not stopped tech companies from continuing to pursue health-tech projects^132^, they are often initiatives that more closely model partnerships with research institutions that do not attempt to access clinical data^133^ or have a direct effect on patients.

This would beg the question of why the design and implementation of effective CAD models cannot occur internally in large research hospitals. While this is a possible route, studies that seek to implement previous research, with a large engineering component, are far less likely to be funded than those that promise novelty^134^. Academic-quality code is also written to be a prototype rather than an end product for users, and so best software engineerings practices, such as version control, documentation, scalability, maintenance, and QA testing, are rarely practiced, though frameworks^135^ and checklists (like the Checklist for Artificial Intelligence in Medical Imaging^136^) for the development and rollout of such products in a clinical environment have been proposed. This would make any academic product built in a hospital potentially unreliable in practice, both from a user experience standpoint and algorithmically, which would only harm clinician trust in the long term.

Additionally, junior AI researchers are in high demand elsewhere^137^, and both market incentives and long-term career prospects of junior biomedical researchers^138^ tend to drive such talent away from these institutions. Large research hospitals, which have positions often funded by federal grants, limit the amounts they pay junior researchers, while tech companies self-fund such positions and are thus able to pay true market rates for top talent. Lack of a centralized database would prevent an objective analysis of this issue^139^, but even a cursory look at salaries in the United States reveals large disparities between tech companies and research hospitals. Base pay of a postdoctoral researcher in AI at Google, Meta, and Microsoft was reported to be $146,787^140^, $140,007^141^, and $148,472^142^, respectively, while the postdoctoral salaries at major research hospitals in the U.S., including Massachusetts General Hospital, the Mayo Clinic, and Johns Hopkins, are based on the NIH stipend guidelines and were reported to be $53,760^143^, $57,923^144^, and $56,369^145^, respectively. This feeds into indirect problems as well, such as poor IT infrastructure at such institutions to support researchers in big data projects, with EHR databases for secondary research access typically designed with small-scale clinical studies in mind^146^, causing researchers to spend time on workarounds to access this data^147^. These forces essentially incentivize AI expertise to move away from workplaces in which real-world medical data is most readily available, hamstring efforts at true translational research. The resultant lack of day-to-day interaction between AI talent and clinicians acts as an additional barrier to implementation.

## Future perspectives

Widespread implementation of neuroimaging CAD models may begin with the limited rolling out of models for the identification of localized structural disorders within large and mid-sized research hospitals, trained only on local data^11,148^. This is a favorable place to start with the deployment of neuroimaging CAD models for three reasons: (1) segmentation-based models are easier to verify by humans than end-to-end diagnostic models, thus helping to catch problems with the models early on; (2) because they make a segmentation rather than a certain diagnosis, they do not need to operate perfectly and thus avert the risk of patient harm due to a false diagnosis, but may be used to notify clinicians to possible high-risk cases in routinely collected data earlier than normal (or, such models may be used to inform a clinician in a diagnosis, e.g. by detecting a rare type of lesion); and (3) by operating within a single institution, they avoid many of the problems associated with site differences.

The rolling out of models on a gradual scale is also important because it would provide clinicians and local technicians with essential training in the use of such models, providing a possible pathway to the future rolling out of neuroimaging CAD models for neurological and, possibly, psychiatric disorders, across many such hospitals. Frequent communication between data scientists and software engineers developing such models and radiologists and other clinicians using them would also encourage a development loop that is useful for creating strong software products^149^. This may happen in the context of either close partnerships between research institutions and tech companies, or within research institutions if internal software development teams could be grown.

This would also help in creating a foundation for more complex CAD models. The implementation of CAD models for psychiatric diagnoses and very early detection in neonates has huge potential, but it would require significantly more investment from hospitals (i.e., in more hardware, data collection procedures, and analysis methods) than those described above. On the side of CAD model development, researchers ought to be incentivized to focus on clinical need, translation, and usefulness rather than the novelty and technical complexity of their methods.

In conclusion, the implementation of CAD models for neuroimaging is hampered by technical, disease-oriented, and institutional challenges, as well as mixed incentives in research and the broader workforce. The likeliest route to the clinical translation of CAD models is the local rolling out of AI that practically aids the workflow of radiologists, in environments in which data scientists and researchers can closely collaborate with clinicians.

### Reporting summary

Further information on research design is available in the Nature Research Reporting Summary linked to this article.

## Supplementary information

Reporting summary
